# Public Grants for Voluntary Hospitals

**Published:** 1910-11-26

**Authors:** 


### PUBLIC GRANTS FOR VOLUNTARY HOSPITALS.

### III.—PRACTICABLE FORMS OF SUBSIDY.

It has been stated that a great opportunity was missed at a time when co-operation on a *per capita* basis might have been possible between the Guardians of the Poor and the voluntary hospitals. The country is now covered with institutions for the sick directly under the control of these functionaries, and a sharp dividing line exists between the voluntary and the rate-supported ideals of medical relief. Looking on the one picture and then on the other, the public may form a judgment as to the extent of their loss when the spirit of private benevolence was carefully subtracted from the care of the aged, the imbecile, and the sick whose misfortunes had labelled them "paupers." It is of enormous importance to the future of the country that in the approaching re-organisation of the existing agencies for dealing with poverty the element of voluntary service shall be restored to its proper place in the treatment of the suffering. It is becoming intolerable that the precautions and alleviations introduced into the hospital wards as the outcome of scientific discoveries should be completely ignored in the workhouse sick wards. But there can be no denying the fact which has been pressed home through the last eighty years, that to thrust upon men compulsorily the onus of caring for their sick neighbours actually hardens their hearts against suffering, while to undertake it spontaneously leads to some of the happiest influences upon character which life affords. We cannot afford to ignore these psychological distinctions. The secret of Poor Law failure lies hidden among them.

In the future, then, much may be gained for the sick and much for the voluntary hospitals if good heed be given to the safeguarding of that priceless treasure which has been defined as "enthusiasm for humanity." For the moment the direction in which local authorities may be best approached in the interests of voluntary hospitals is for the removal of the old grievance of the rates. It has been seen that in several of the American States the simple and very practical course has been taken of subsidising private hospitals by releasing them from their obligations as ratepayers. This measure of relief would in many instances suffice to wipe out the burdensome yearly deficit. In the accounts of the London Hospital for 1908 the rates stand for a total of £1,452. And it is interesting to see that the total in small sums subscribed by the working people of the locality through the agency of the Hospital Saturday Fund and the workmen's collections together did not equal this amount. Remission of rates has the advantage that it is a gracious recognition by public authorities of the public benefits conferred by the hospital, yet it carries with it no responsibility for management, and no obligation to participate in the burdens of the voluntary supporters. It is a measure which has long been urged and is so entirely reasonable that it can hardly fail of accomplishment in the near future.

The question of State grants in aid of the hospitals is a far more complicated one. The American system through which grants are made to voluntary hospitals after investigation and in response to compliance with a code of rules drawn up by a State board of charities has much to recommend it, and has already, as has been shown, been initiated in London on a purely voluntary basis. The King's Fund stands as an object-lesson in the methods through which outside support might be accorded to the voluntary hospitals of the kingdom without weakening their independence or alienating public charity. Nothing is more valuable in this connection than the successive records supplied by Sir Henry Burdett in his "Hospital Annual," tracing the extent to which the volume of private benevolence has swelled with the growth of the King's Fund. It may be anticipated that State grants bestowed on the same lines, so that not London hospitals alone might participate in the encouragement afforded by a central fund of this description, would exercise a corresponding influence all over the country in stimulating private charity.

In view of the ever-increasing costliness of medical and nursing treatment, if it is to be effectual, some State encouragement of the services rendered by voluntary hospitals would seem to be rising into the front rank among questions of national importance. The more strongly we hold to the necessity for the voluntary principle in relieving the necessities of the sick, the more earnestly will some solution be sought for the gathering difficulties of the hospital financier. A *per capita* basis is not the ideal one under English hospital conditions. Unless the payment is considerably less than the cost of the patient, the voluntary principle is lost, and under any circumstances difficulties of administration arise, and a condition of divided responsibility for the financial stability of the institution is set up. It is very easy, moreover, to discourage the flow of subscriptions when it is once understood that only a certain proportion of the income is derived from charity.

These objections do not apply to subsidies in aid of restorations, rebuilding and extension. The onus of providing the enormous capital sums required for building schemes has often in the past pressed with such crushing weight on a community that when all has been accomplished, and the new institution or wing is ready for the patients, the money for upkeep has proved unattainable. This was the case with the Clarence Wing at St. Mary's Hospital in London, and is happening at St. Mary's, Manchester. The instances, indeed, are too numerous to quote, and in several towns at this moment are creating widespread disappointment and perplexity. Subsidies from the State towards the outlay on new buildings would act as a powerful stimulus to private zeal in hospital matters. The finely planned institution, representing the highest point attainable in hospital construction and fitting would, it may be safely predicted, act as a valuable object-lesson in the treatment of the sick, and would prove a worthy gift from the State to localities where Imperialism at the present time spells taxation pure and simple, and the glories of participation in national magnificence read as mere newspaper fairy tales.

The extraordinary expenses of the hospitals—London, Provincial, Scottish, and Irish—during the year 1907 amounted to £516,146, chiefly for building and restoration purposes. The expenditure under all headings for the same period amounted to £3,442,021, about six and a half times the total for building alone.

It may be seen, therefore, what heavy demands are made upon charitable persons by bricks and mortar, and what a striking measure of encouragement would be extended to voluntary hospital work if State support could be enlisted in this direction.

